# Comparative Chemical Profiles of Essential Oils and Hydrolate Extracts from Fresh Flowers of Eight *Paeonia suffruticosa* Andr. Cultivars from Central China

**DOI:** 10.3390/molecules23123268

**Published:** 2018-12-10

**Authors:** Gaoming Lei, Jie Li, Tao Zheng, Junqiao Yao, Jingjing Chen, Lengxin Duan

**Affiliations:** 1 Department of Pharmaceutical Sciences, Medical College, Henan University of Science and Technology, Luoyang 471023, China; lijie@haust.edu.cn (J.L.); 170317200708@stu.haust.edu.cn (J.C.); lengxinduan@haust.edu.cn (L.D.); 2 National Flower Garden Administration of Luoyang, Luoyang 471022, China; tony7811@163.com; 3 Department of Scientific Research, Peony Institute of Luoyang, Luoyang 471022, China; mdsyjq@163.com

**Keywords:** *Paeonia suffruticosa* Andr., essential oil, hydrolate extract, chemical profile, GC-MS, GC-FID

## Abstract

*Paeonia suffruticosa* Andr. is a famous ornamental and aromatic plant with hundreds of cultivars in China. The objective of this work was to investigate comparative chemical profiles of essential oils and hydrolate extracts from eight *P. suffruticosa* Andr. cultivars from Central China. The percentages of hydrocarbons in hydrolate extracts (≤1.1%) were significantly lower than those in the essential oils (29.8–63.7%). The percentages of oxygenated compounds in hydrolate extracts (98.3–99.8%) were significantly higher than those in the essential oils (34.8–69.6%). Multivariate analyses with hierarchical clusters and principal components further indicated the chemical differences between essential oils and hydrolate extracts. Due to predominance of oxygenated compounds and almost trace level of hydrocarbons, *P. suffruticosa* Andr. hydrolate extracts could be good alternatives to the essential oils. Moreover, distribution of major oxygenated compounds in hydrolate extracts varied with cultivars. Hydrolate extracts from ’SHT’, ’WLPS’ and ’BXT’ presented chemotypes of methylated phenols (65.0%), 2-phenylethanol (64.4%) and geraniol + citronellol + nerol (59.9%), respectively. Those from five other cultivars presented somewhat mixed chemotypes. These results were further confirmed by quantitative evaluation relative to the major oxygenated compounds. The outcome of this work will promote applications of *P. suffruticosa* Andr. hydrolate extracts in fragrances and cosmetics.

## 1. Introduction

*Paeonia suffruticosa* Andr. is a deciduous shrub in the *Moutan* section, genus *Paeonia* and family Ranunculaceae. It is a famous ornamental and aromatic plant native to China and is widely cultivated throughout the country. The plant grows up to 2 m tall and flowers during April and early May, emitting a pleasant floral aroma. The flowers are solitary in terminal and are 10–17 cm wide. The petals are single or double, in red, red-purple, pink or white colors. Meanwhile, petals are obovate 5–8 cm long and 4.2–6 cm wide, with the apex irregularly incised. This species has been cultivated for over 1000 years in China and there are now hundreds of cultivars within the country. Specifically, Luoyang (Henan Province) and Heze (Shandong Province) are two important places of cultivation [1].

Up to now, studies regarding fresh flowers of *P. suffruticosa* Andr. mostly focused on emitted volatiles, using headspace/gas chromatography–mass spectrometry (HS/GC-MS) or headspace–solid phase micro-extraction (HS-SPME)/GC-MS techniques [2,3,4,5]. The floral volatiles from different cultivars mainly include (*Z*)-β-ocimene, α-pinene, citronellol, linalool, geraniol, neryl acetate, 2-phenylethanol, 1,3,5-trimethoxybenzene, 2,3-dihydroxypropanal, 3-methylbutanol, 2-ethylhexanol, (*Z*)-3-hexen-1-ol, 1-hexanol and pentadecane [2,3,4,5]. Results from different studies vary considerably, most probably due to variations in cultivars [2,4], flowering stages [5] and analytical conditions.

Products derived from flowers of *P. suffruticosa* Andr. include the concrete [6,7], absolute [8,9,10] and essential oil [10,11,12]. Among these the essential oil is most valued and used in perfumes and cosmetics [10,11,12]. It should be noted that only the product obtained by hydro-, steam or dry distillation (may be combined with various sample pretreatments [13,14,15] or substrates addition [14,16], or assisted with mass-transfer improving techniques [14,17,18]) can be termed as ‘essential oil’ (with the exception of cold expression for citrus fruits) [19]. The main essential oil components of certain *P. suffruticosa* Andr. cultivars are heptadecane, heneicosane, 1,3,5-trimethoxybenzene, *cis*-linalool oxide (furanoid), *trans*-linalool oxide (furanoid) and α-terpineol, etc [10,11,12].

Hydrolate extract is the fraction recovered from the aqueous distillate (hydrolate) generated during hydro- or steam distillation. It is also known as recovered essential oil or water-soluble essential oil. It is mainly composed of volatile compounds due to its isolation through distillation-extraction process. In the cases of *Lavandula angustifolia* Mill. [20], *Tagetes minuta* L. [21], *Osmanthus fragrans* Lour. [22], *Cymbopogon flexuosus* (Nees *ex* Steud.) Wats. [23], *Daucus muricatus* L. [24], *Calendula arvensis* L. [25], and certain species in genera *Yulania* [26,27], *Cerasus* [28] and *Rosa* [29], hydrolate extracts are composed of relatively high percentages of oxygenated compounds and low percentages of alkanes and terpene hydrocarbons. Oxygenated compounds such as alcohols, ethers, ketones, aldehydes and esters are preferable for their aroma or flavor characteristics. Therefore higher percentage of these compounds corresponds to higher organoleptic quality [27,30]. On the other hand, alkanes and terpene hydrocarbons are undesired compounds for their poor contribution to the aroma or flavor [29,30]. Moreover, terpene hydrocarbons tend to undergo oxidation, hydrolysis or polymerization when exposed to air or light, inducing decline in essential oil quality [30].

In spite of the existence of hundreds of cultivars, studies on essential oils of *P. suffruticosa* Andr. are limited to just a few ones. Moreover, to the best of our knowledge, there are no studies on chemical compositions of hydrolate extracts or comparative compositions of essential oils and hydrolate extracts from different *P. suffruticosa* Andr. cultivars. Therefore the objective of this work was to investigate comparative chemical profiles of essential oils and hydrolate extracts of eight *P. suffruticosa* Andr. cultivars from Central China. GC-MS and GC–flame ionization detector (GC-FID) were used to analyze the samples. Multivariate tools of hierarchical cluster analysis (HCA) and principal component analysis (PCA) [31] were employed to further evaluate the chemical variation among samples. Chemical compositions of essential oils and hydrolate extracts were compared. The importance of hydrolate extracts from different cultivars was evaluated. The outcome of this work can contribute to applications of *P. suffruticosa* Andr. hydrolate extracts in fragrances and cosmetics.

## 2. Results and Discussion

### 2.1. Yields of Essential Oils and Hydrolate Extracts

Hydro-distillation is the preferred method for essential oil extraction according to the Chinese [32] and European [33] Pharmacopoeias. In order to improve the extraction efficiency, salt-mediated ultrasound-assisted hydro-distillation was employed in this study. Immersion of plant materials into sodium chloride solution of a suitable concentration leads to changes of osmotic pressure of plant cells and promotes cells rupture, which accelerates the release of essential oil from inside the cells [16]. Meanwhile, ultrasonic processing improves rupture of cell walls through collapse of cavitation bubbles, enhances solvent penetration and facilitates release of essential oil [13,14]. Consequently it was expected that a synergistic effect could be generated through combination of the two. Key operating parameters of salt-mediated sonication include ultrasonic power and frequency, concentration of sodium chloride solution and treatment duration. Based on preliminary tests, the optimal pretreatment condition was selected as ultrasonic power of 400 W and frequency of 40 kHz, sodium chloride concentration of 5% (*w*/*v*) and treatment duration of 30 min.

The yields of essential oils of *P. suffruticosa* Andr. varied with cultivars from 0.28% to 0.93% whereas those of hydrolate extracts varied from 0.25% to 0.83% (Table 1). As it turned out, cultivar ‘SHT’ possessed the highest yields for both essential oil and hydrolate extract whereas ‘HH’ possessed the lowest ones.

Studies on *P. suffruticosa* Andr. essential oils up to now are limited to a few cultivars [10,12], particularly ’FDB’ which is mainly planted for pharmaceutical uses of its cortex [34] or extraction of seed oil [10]. The essential oil yield from ’FDB’ in this study was higher than those obtained by conventional hydro [12] or steam distillation [10]. As mentioned, this could be due to synergy of osmosis and cavitation effects generated during salt-mediated sonication. In addition, the essential oil yields of *P. suffruticosa* Andr. flowers in this study were of the same order of magnitude as that of *P. suffruticosa* Andr. buds (cultivar unknown) [11].

### 2.2. Comparative Chromatographic Profiles of Essential Oils and Hydrolate Extracts

**Figure 1:**
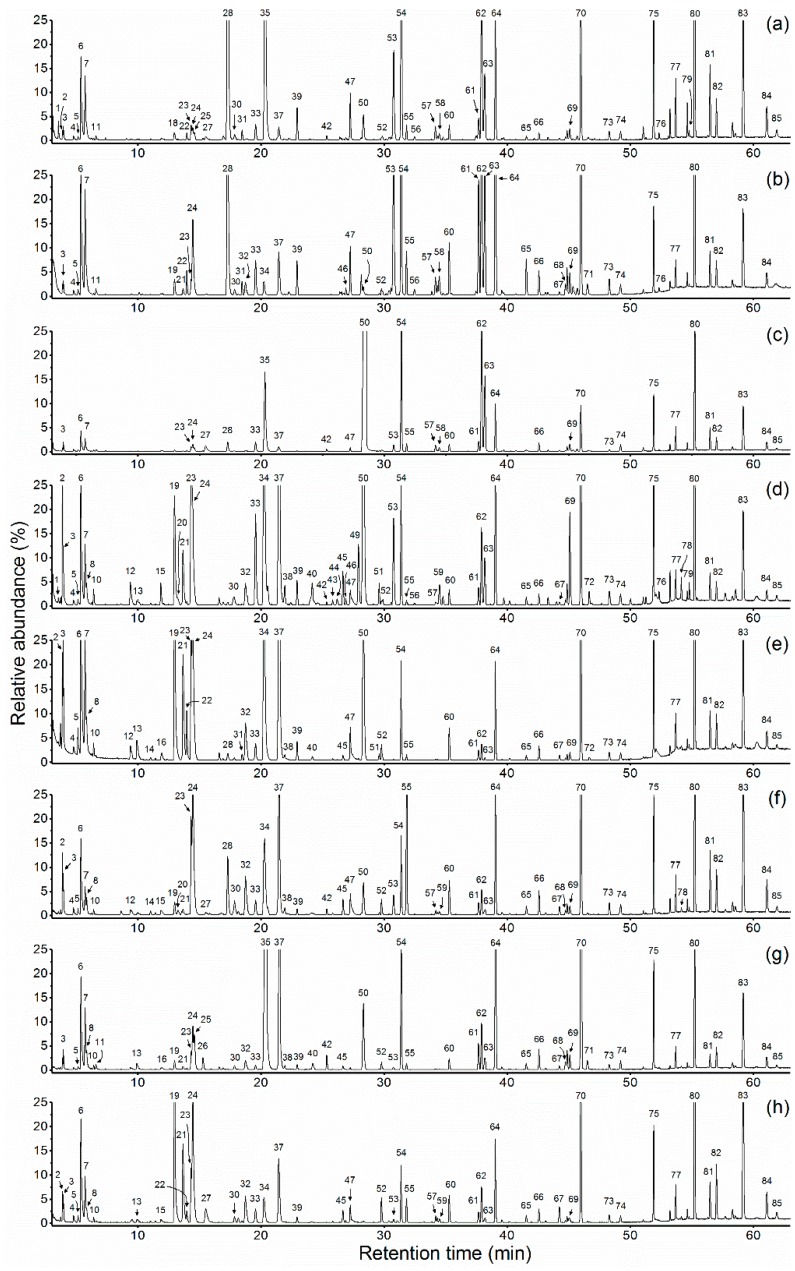
Chromatographic profiles of essential oils of eight *Paeonia suffruticosa* Andr. cultivars: (**a**) ‘JYH’; (**b**) ‘JXQ’; (**c**) ‘SHT’; (**d**) ‘FDB’; (**e**) ‘HH’; (**f**) ‘YH’; (**g**) ‘BXT’; (**h**) ‘WLPS’.

**Figure 2:**
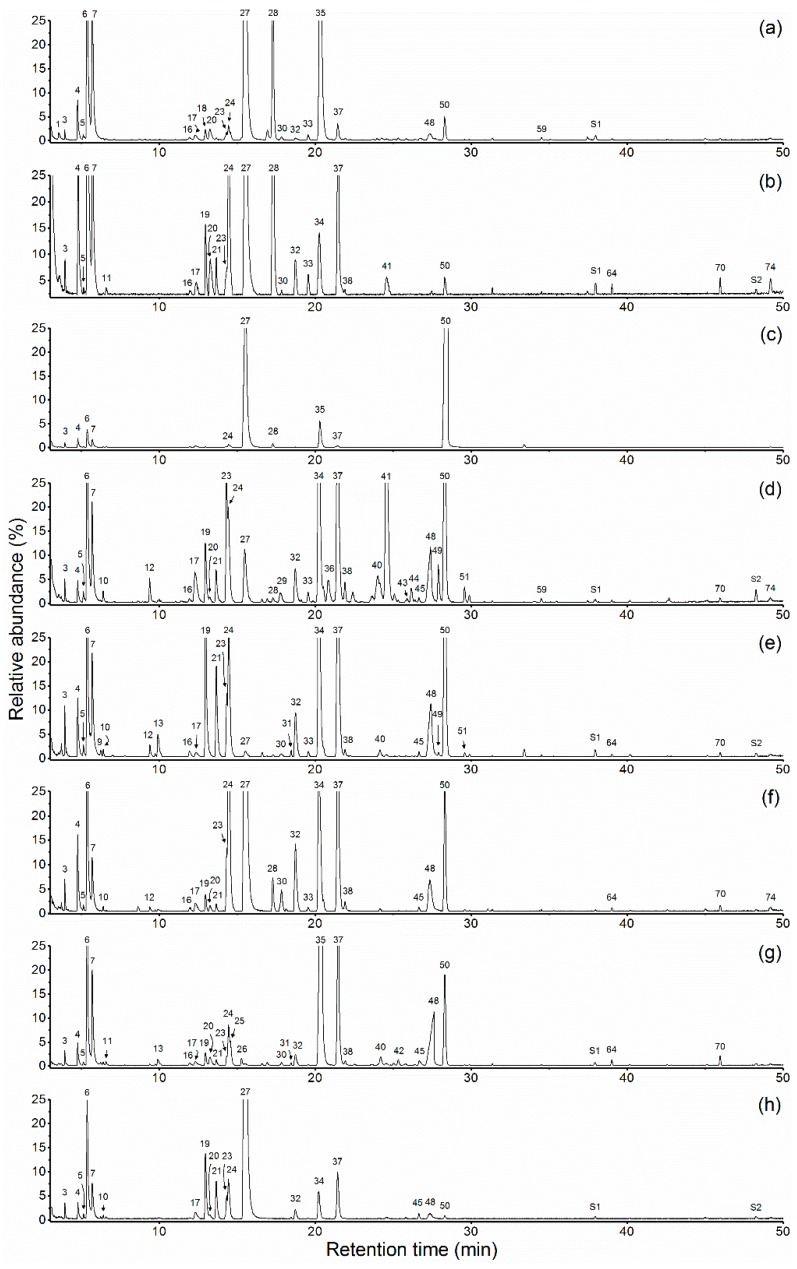
Chromatographic profiles of hydrolate extracts of eight *Paeonia suffruticosa* Andr. cultivars: (**a**) ‘JYH’; (**b**) ‘JXQ’; (**c**) ‘SHT’; (**d**) ‘FDB’; (**e**) ‘HH’; (**f**) ‘YH’; (**g**) ‘BXT’; (**h**) ‘WLPS’; S1, triethyl citrate; S2, dibutyl phthalate.

Chemical compositions of essential oils and hydrolate extracts of *P. suffruticosa* Andr. cultivars are given in Table 2. Typical chromatographic profiles are given in Figure 1 and Figure 2. Altogether 85 compounds were identified accounting for 97.7–99.8% of the total composition.

Significant compositional differences were observed between essential oils and hydrolate extracts. Hydrocarbons accounted for considerable proportions (29.8–63.7%) of the essential oil components but their percentages in hydrolate extracts were rather low (≤1.1%). On the other hand, oxygenated compounds predominated (98.3–99.8%) in hydrolate extracts. The difference between essential oils and hydrolate extracts can be explained by solubility effect. Oxygenated compounds possess relatively high water solubility and tend to dissolve into the hydrolate during distillation [21,22,23]. Besides, hydro-distillation employing Clevenger apparatus as a pharmacopoeia method recovers only part of the water-soluble essential oil components [21,23,27].

The percentages of non-terpene hydrocarbons in essential oils (29.2–57.1%) were much higher than those in hydrolate extracts (≤1.1%). These in essential oils were mainly tricosane (3.6–14.2%), nonadecane (2.1–13.0%), heptadecane (2.1–11.7%), pentadecane (2.1–11.2%), pentacosane (2.0–9.1%) and (*E*)-8-heptadecene (≤4.1%). These were roughly consistent with literature results that *n*-alkanes such as heptadecane and heneicosane account for relatively high proportions of essential oil components of *P. suffruticosa* Andr. [11,12].

Non-terpene hydrocarbons are undesirable for their poor contribution to the aroma or flavor of essential oils [29]. Essential oils of *P. suffruticosa* Andr. also contained certain amounts of terpene hydrocarbons (0.1–6.6%), which were not detected in the hydrolate extracts. It is known that oxidation or polymerization of terpene hydrocarbons which occur under oxygen or light is the main reason for deterioration of essential oil quality [30].

The percentages of non-terpene oxygenated compounds in hydrolate extracts (24.2–96.6%) were significantly higher than those in essential oils (11.7–62.5%) from the same cultivars. These in hydrolate extracts were mainly 2-phenylethanol, 1,3,5-trimethoxybenzene, (*Z*)-3-hexen-1-ol, 1-hexanol, 1,4-dimethoxybenzene and cinnamyl alcohol, etc. 2-Phenylethanol was present with the highest percentage in hydrolate extract from ‘WLPS’ (64.4%) whereas it was not detected in that from ‘BXT’. This compound also predominates in rose (*Rosa damascena* Mill. and *R. rugosa* Thunb.) hydrolates [29,35]. In addition, it occurs in floral volatiles of certain *P. suffruticosa* Andr. cultivars [4]. 1,3,5-Trimethoxybenzene existed with the highest percentage in hydrolate extract from ’SHT’ (64.7%). It is a key component responsible for specific floral scent of Chinese rose (*R. chinensis* Jacq.) [36] and also occurs in floral volatiles of several *P. suffruticosa* Andr. cultivars [2]. This compound is an effective sedative and can also be used as a cosmetic additive [36]. Another methylated phenol was 1,4-dimethoxybenzene. It is also identified as a key floral scent component from willows (*Salix* species) that attracts female bees (*Andrena vaga* Panz.) [37]. With regard to C6 aliphatic alcohols, (*Z*)-3-hexen-1-ol and 1-hexanol are also found to be characteristic volatile components emitted from *P. suffruticosa* Andr. buds [5].

The percentages of oxygenated terpenes in hydrolate extracts were higher than those in essential oils for cultivars except for ‘SHT’ and ‘WLPS’. These were mainly monoterpene alcohols, particularly geraniol, citronellol, nerol, linalool and linalool oxides (furanoid). Geraniol, citronellol and nerol are recognized as major monoterpene alcohols characteristic of rose oil [29]. In addition, linalool oxides (furanoid) are reported to predominate in hydrolate volatiles of *Osmanthus fragrans* Lour. [22]. Other oxygenated terpenes in hydrolate extracts included geranic acid, which was not detected in the essential oils. Geranic acid also occurs in lemongrass (*Cymbopogon citratus* (DC.) Stapf) and is the key substance responsible for potent tyrosinase inhibition activity [38].

**Figure 3:**
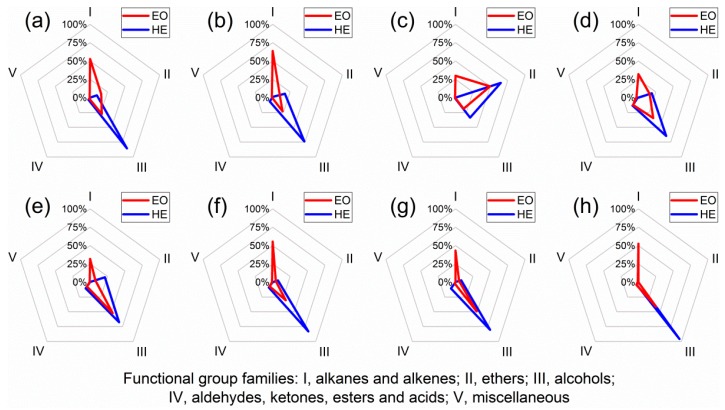
Comparative chemical profiles regarding functional group families of essential oils and hydrolate extracts from eight *Paeonia suffruticosa* Andr. cultivars: (**a**) ‘JYH’; (**b**) ‘JXQ’; (**c**) ‘SHT’; (**d**) ‘FDB’; (**e**) ‘HH’; (**f**) ‘YH’; (**g**) ‘BXT’; (**h**) ‘WLPS’; EO, essential oil; HE, hydrolate extract.

Chemical profiles were further compared in terms of functional group families (Figure 3). Briefly, the hydrolate extracts possessed higher percentages of alcohols and much lower percentages of alkanes and alkenes in comparison with essential oils from the same cultivars. For ‘YH’, ‘WLPS’, ‘JXQ’, ‘BXT’ and ‘HH’, the essential oils were mainly composed of alkanes and alkenes (31.8–63.7%) and alcohols (22.8–52.4%). For ‘FDB’, ‘SHT’ and ‘JYH’, the essential oils were mainly composed of alkanes and alkenes (29.8–52.9%), alcohols (18.8–34.5%) and ethers (16.0–49.7%). On the other hand, for ‘WLPS’, ‘JYH’, ‘YH’ and ‘BXT’, the hydrolate extracts were predominantly composed of alcohols (80.4–95.6%). For ‘JXQ’, ‘HH’, ‘FDB’ and ‘SHT’, the hydrolate extracts were mainly composed of alcohols (33.9–73.7%) and ethers (17.6–65.0%).

### 2.3. Multivariate Aspects

In order to further evaluate comparative chemical profiles of essential oils and hydrolate extracts, multivariate analyses with HCA and PCA were performed.

**Figure 4:**
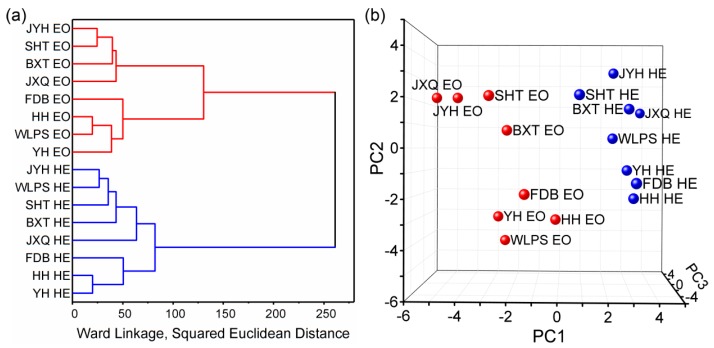
Multivariate analysis for essential oils and hydrolate extracts of eight *Paeonia suffruticosa* Andr. cultivars: (**a**) HCA dendrogram; (**b**) PCA score plot across dimensions of the first three principal components; EO, essential oil; HE, hydrolate extract.

Clustering of observations in HCA serves as an unsupervised method being able to classify observations into groups which are initially unknown [31]. As shown in Figure 4a, all samples can be clearly grouped into two clusters: one corresponding to the essential oils from eight cultivars and the other for the hydrolate extracts. Meanwhile, the distance between essential oils and hydrolate extracts was larger than those for essential oils or hydrolate extracts among cultivars. These indicated that the essential oils and hydrolate extracts of *P. suffruticosa* Andr. presented different chemical characteristics.

PCA is another unsupervised approach and is able to reduce the dimensionality of multivariate data [31]. The first three principal components altogether accounted for 62.3% of the total variability and therefore can basically describe the chemical variation among samples. As shown in Figure 4b, the essential oils and hydrolate extracts were separated from each other. Besides, all essential oil samples had negative values along the first principal component (PC1) whereas hydrolate extracts had positive values along PC1. All these indicated that the essential oils and hydrolate extracts of *P. suffruticosa* Andr. possessed significantly different chemical profiles. The results were consistent with those of HCA.

### 2.4. Importance of P. Suffruticosa Andr. Hydrolate Extracts

As revealed, hydrolate extracts of eight *P. suffruticosa* Andr. cultivars were characterized by a predominance of oxygenated compounds and almost trace levels of hydrocarbons. Since a higher percentage of oxygenated compounds corresponds to higher organoleptic quality [30], *P. suffruticosa* Andr. hydrolate extracts could be good alternatives to the essential oils. Typical oxygenated compounds were 2-phenylethanol, geraniol, citronellol, nerol, 1,3,5-trimethoxybenzene, 1,4-dimethoxybenzene, (*Z*)-3-hexen-1-ol, 1-hexanol, linalool and linalool oxides. Most of them are associated with the natural floral scent of *P. suffruticosa* Andr. [2,3,4,5]. Experimental mass spectra of some representative components are given in Figure S1 in the Supplementary Materials.

**Figure 5:**
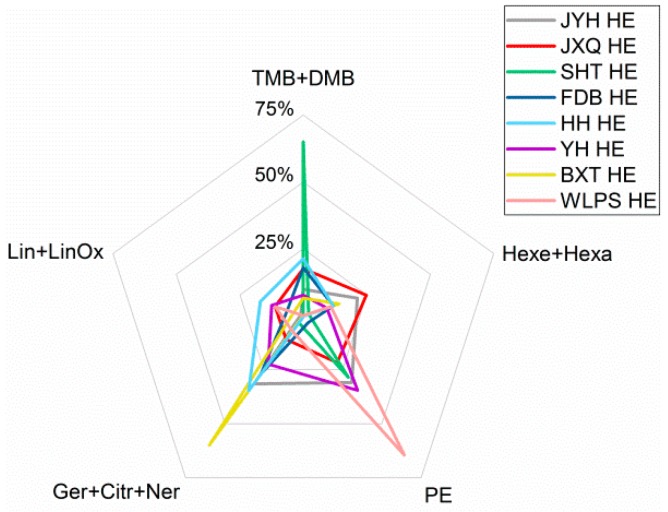
Distribution of typical oxygenated compounds in hydrolate extracts of different *Paeonia suffruticosa* Andr. cultivars. TMB, 1,3,5-trimethoxybenzene; DMB, 1,4-dimethoxybenzene; Hexe, (Z)-3-hexen-1-ol; Hexa, 1-hexanol; PE, 2-phenylethanol; Ger, geraniol; Citr, citronellol; Ner, nerol; Lin, linalool; LinOx, linalool oxides; HE, hydrolate extract.

Furthermore, distribution of major oxygenated compounds in hydrolate extracts varied with cultivars. As shown in Figure 5, hydrolate extract from cultivar ‘SHT’ presented chemotype of methylated phenols (65.0%). This made it possess an organoleptic quality similar to Chinese rose oil. Hydrolate extract from ‘WLPS’ presented chemotype of 2-phenylethanol (64.4%). This allowed it to be an alternative to less productive rose hydrolate in China. Further, hydrolate extract from ‘BXT’ presented chemotype of geraniol + citronellol + nerol (59.9%) and, owing to very low percentage of hydrocarbons (0.5%), it could be a good alternative to expensive rose oil. On the other hand, hydrolate extracts from five other cultivars presented somewhat mixed chemotypes.

The hydrolate extracts were further evaluated quantitatively relative to the major oxygenated compounds. The content of 1,3,5-trimethoxybenzene in hydrolate extract of ‘SHT’, that of 2-phenylethanol in hydrolate extract of ‘WLPS’ and that of citronellol in hydrolate extract of ’BXT’ were 596.4, 590.1 and 410.6 mg/g, respectively (Table 3). These were basically consistent with the qualitative results. Consequently, hydrolate extracts of *P. suffruticosa* Andr. from different cultivars could find uses in fragrances and cosmetics.

## 3. Materials and Methods

### 3.1. Plant Materials and Chemicals

Fresh flowers of *P. suffruticosa* Andr. were collected at full bloom stage in April, from the National Flower Garden (137 m altitude, 34°39″ N latitude and 112°27″ E longitude), Luoyang, China. Altogether eight cultivars were collected, namely ‘JYH’ (‘JuanYeHong’), ‘JXQ’ (‘JinXiuQiu’), ‘SHT’ (‘ShanHuTai’), ‘FDB’ (‘FengDanBai’), ‘HH’ (‘HuHong’), ‘YH’ (‘YaoHuang’), ‘BXT’ (‘BaiXueTa’) and ‘WLPS’ (‘WuLongPengSheng’). The plant materials were identified by Professor Huanling Zhang, Peony Institute of Luoyang. Voucher specimens are deposited at the Herbarium of Department of Pharmaceutical Sciences, Henan University of Science and Technology. The water contents of fresh flowers were determined by distillation with toluene [39]. Analytical standards of 2-phenylethanol, 1,3,5-trimethoxybenzene, geraniol and citronellol were supplied by Aladdin Chemistry Co., Ltd. (Shanghai, China). Mixed standard of *n*-alkanes C7-C30 (1000 μg/mL for each alkane component in hexane) from Supelco (Bellefonte, PA, USA) was used as received. Deionized water was prepared with Millipore Direct-Q system. Other reagents were of analytical grade.

### 3.2. Extraction of Essential Oils and Preparation of Hydrolate Extracts

*P. suffruticosa* Andr. fresh flowers (800 g) mixed with 5% (*w/v*) sodium chloride solution (1800 mL) were sonicated using a KQ-500GDV ultrasonator (Kunshan Ultrasonic Instruments Co., Ltd., Kunshan, China) at power of 400 W and frequency of 40 kHz for 30 min. The mixture was then hydro-distilled for 2 h with essential oil trapped in a Clevenger type apparatus [32]. The essential oil collected was diluted with hexane to approximately 10 mL. It was then dehydrated with anhydrous sodium sulfate, filtered and evaporated to afford the neat oil. It was accurately weighed and stored at −5 °C.

*P. suffruticosa* Andr. fresh flowers (800 g) mixed with 5% (*w*/*v*) sodium chloride solution (1800 mL) were sonicated under identical conditions (power, frequency and duration) as those for essential oil extraction. After that, hydro-distillation was performed for 2 h to afford the hydrolate (approximately 400 mL). Sodium chloride (40 g) was dissolved into the hydrolate and the resulting solution was immediately extracted with methylene chloride (40 mL aliquots and five cycles). The organic layer was dehydrated with anhydrous sodium sulfate, filtered and evaporated to yield the hydrolate extract which was an oily liquid. It was accurately weighed and stored at −5 °C.

All the extraction processes were performed in triplicate. Yields of essential oils and hydrolate extracts were calculated as *w*/*w* % on dry basis. They are presented as mean ± SD from triplicate extractions.

### 3.3. GC-MS and GC-FID Analysis

The essential oils and hydrolate extracts were diluted at 1:50 (*v*/*v*) with hexane and methylene chloride, respectively. GC-MS was carried out on an Agilent 6890N gas chromatograph coupled with a 5975I mass selective detector (MSD) and fitted with a 7683B series injector. Chromatographic separation was performed on HP-5ms (30.0 m × 0.25 mm i.d., film thickness 0.25 μm). Injector temperature was 250 °C. Injection volume was 1.0 μL with split ratio of 20:1. Helium was used as carrier gas with flow rate of 1.0 mL/min. The column temperature was programmed from 50 °C to 200 °C at 3 °C/min, from 200 °C to 240 °C at 10 °C/min, and held at 240 °C for 10 min. With regard to MSD conditions, temperature of transfer line was 280 °C. The ion source temperature was 230 °C with the electron energy of 70 eV. The MS scan range was 29–400 amu. GC-FID was performed using an Agilent 6890N gas chromatograph equipped with a 7683B auto-sampler and coupled with a FID. A HP-5ms column (30.0 m × 0.25 mm i.d., film thickness 0.25 μm) was used. The chromatographic conditions were the same as those for GC-MS. FID temperature was 280 °C.

Components were identified based on mass spectra and retention indices [40]. Experimental mass spectra were matched with NIST 05a library. Experimental retention indices calculated using retention times of reference *n*-alkanes C7-C30 run under identical conditions were compared with literature data [41,42,43]. Standards of 2-phenylethanol, 1,3,5-trimethoxybenzene, geraniol and citronellol run under the same conditions were also employed for identification. The percentage of each component was calculated according to the following equation:

(1) $$X_{i}=\frac{f_{i}A_{i}}{\sum f_{i}A_{i}}\times 100\%$$

where *X_i_* and *A_i_* are the percentage and GC-FID peak area of individual component, respectively. Parameter *f_i_* is the predicted relative response factor (relative to heptane) for component *i*, calculated based on the approach recommended by IOFI [44]. Percentage compositions are presented as mean value of triplicate extractions and duplicate injections.

### 3.4. Quantitation of Major Components in Hydrolate Extracts

System suitability was tested including resolution, number of theoretical plates, sensitivity, tailing factor and injection repeatability [45]. Accurately weighed hydrolate extracts (approximately 0.1000 g) were diluted appropriately with ethyl acetate and analyzed under the same GC-FID conditions as described in Section 3.3. The major components in the hydrolate extracts can be separated at baseline (*R* > 1.5). In addition, a mixed standard solution of 2-phenylethanol, 1,3,5-trimethoxybenzene, geraniol and citronellol (approximately 2.000 mg/mL for each in ethyl acetate) were analyzed under identical chromatographic conditions and, the number of theoretical plates (> 50,000), signal-to-noise ratio (much higher than 10) and tailing factor (0.9–1.3) for all the five compounds were satisfactory. Moreover, the mixed standard solution was injected (with auto-sampler) in sextuplicate and RSDs of the peak areas for each compound were found to be less than 2.0%.

For quantitation of the major components in hydrolate extracts, the following equation was employed [45]:

(2) $$C_{S}=C_{R}\frac{A_{S}}{A_{R}}$$

where *C*_S_ and *C*_R_ are the concentrations (mg/mL) of target compound in sample solution and reference solution, respectively. *A*_S_ and *A*_R_ are the peak areas of target compound in sample solution and reference solution, respectively. The content (mg/g) of target compound in hydrolate extracts was then calculated as dividing *C*_S_ by the concentration (g/mL) of hydrolate extract in sample solution relative to the dilution process. The contents are presented as mean value of triplicate extractions and duplicate injections.

### 3.5. Statistical Analysis

Statistical analysis was performed with Minitab 17 software (Minitab Inc., State College, PA, USA). Analysis of variance (ANOVA) followed by Tukey’s HSD test was employed to determine statistical significance of variations within data. The significance level was set at 0.05 unless otherwise noted. A data matrix of 16 samples × 31 components was constructed for multivariate analyses. It consisted of percentages of main components and excluded minor components (<2.0% in all samples). HCA was performed using standardized variables with Ward linkage and squared Euclidean distance. PCA was performed using the correlation matrix.

## 4. Conclusions

Essential oils and hydrolate extracts are two important natural products obtained by hydro—or steam distillation from aromatic plants. For species like *P. suffruticosa* Andr. with hundreds of cultivars, it is very necessary to study comparative chemical profiles of essential oils and hydrolate extracts from different cultivars. In this study, eight cultivars from Central China were investigated. Hydrocarbons accounted for considerable proportions of the essential oil components whereas oxygenated compounds predominated in the hydrolate extracts. Multivariate analyses with HCA and PCA further indicated the chemical differences between essential oils and hydrolate extracts. Owing to predominance of oxygenated compounds and almost trace level of hydrocarbons, *P. suffruticosa* Andr. hydrolate extracts could be good alternatives to the essential oils. Typical oxygenated components of the hydrolate extracts were 2-phenylethanol, geraniol, citronellol, nerol, 1,3,5-trimethoxybenzene, 1,4-dimethoxybenzene, (*Z*)-3-hexen-1-ol, 1-hexanol, linalool and linalool oxides. Moreover, hydrolate extracts from different cultivars presented different chemotypes, namely methylated phenols, 2-phenylethanol, geraniol + citronellol + nerol, and mixed types. These were further confirmed by quantitative evaluation relative to the major oxygenated components. The outcome of this work will contribute to applications of *P. suffruticosa* Andr. hydrolate extracts in fragrances and cosmetics.

## Figures and Tables

**Table 1:** Yields of essential oils and hydrolate extracts from fresh flowers of eight *Paeonia suffruticosa* Andr. cultivars.

Cultivars ^a^	Water Contents (%) ^b^	Yields on Dry Basis (*w/w*%) ^c^	
		Essential Oil	Hydrolate Extract
‘JYH’ (‘JuanYeHong’)	78	0.45 ± 0.10	0.49 ± 0.12
‘JXQ’ (‘JinXiuQiu’)	78	0.30 ± 0.07	0.35 ± 0.08
‘SHT’ (‘ShanHuTai’)	77	0.93 ± 0.15	0.83 ± 0.14
‘FDB’ (‘FengDanBai’)	79	0.56 ± 0.09	0.52 ± 0.11
‘HH’ (‘HuHong’)	80	0.28 ± 0.06	0.25 ± 0.08
‘YH’ (‘YaoHuang’)	81	0.47 ± 0.11	0.60 ± 0.15
‘BXT’ (‘BaiXueTa’)	80	0.50 ± 0.12	0.48 ± 0.10
’WLPS’ (‘WuLongPengSheng’)	79	0.33 ± 0.08	0.52 ± 0.13

^a^ Abbreviated cultivar names followed by corresponding full names in parentheses; ^b^ Water contents were determined by distillation with toluene; ^c^ Yields are presented as mean ± SD from triplicate extractions.

**Table 2:** Chemical compositions (%) of essential oils and hydrolate extracts from fresh flowers of eight *Paeonia suffruticosa* Andr. cultivars. ^a^

No.	RI_exp _^b^	RI_lit _^c^	Components	Essential Oils								Hydrolate Extracts							
				JYH	JXQ	SHT	FDB	HH	YH	BXT	WLPS	JYH	JXQ	SHT	FDB	HH	YH	BXT	WLPS
1	−	768	1-Hexen-3-one	0.3	nd ^d^	nd	tr ^e^	nd	nd	nd	nd	0.3	nd	nd	nd	nd	nd	nd	nd
2	800	800	Octane	0.1	tr	nd	1.6	0.9	0.9	tr	0.5	nd	nd	nd	nd	nd	nd	nd	nd
3	802	802	Hexanal	0.3	0.2	0.3	0.7	1.8	0.9	0.6	0.8	0.2	0.6	0.2	0.4	0.8	0.7	0.5	0.6
4	837	836	Furfural	tr	tr	nd	tr	tr	0.2	nd	0.3	1.5	4.9	0.7	0.6	1.8	2.4	1.1	0.9
5	853	854	(*E*)-2-Hexenal	tr	tr	nd	tr	0.5	0.2	tr	0.2	tr	tr	nd	0.3	0.3	tr	tr	tr
6	863	858	(*Z*)-3-Hexen-1-ol	2.6	4.0	1.1	2.8	6.2	3.0	3.3	5.0	11.9	17.2	1.4	8.2	7.6	6.9	8.7	7.9
7	877	874	1-Hexanol	2.4	3.6	0.8	1.3	3.1	1.1	2.1	2.1	9.4	7.6	0.7	3.6	4.2	2.4	5.5	2.9
8	882	882	2,6-Dimethyl-1,5-heptadiene	nd	nd	nd	0.4	1.0	0.5	0.5	0.5	nd	nd	nd	nd	nd	nd	nd	nd
9	900	900	Nonane	nd	nd	nd	nd	nd	nd	nd	nd	nd	nd	nd	nd	0.1	nd	nd	nd
10	904	903	Heptanal	nd	nd	nd	0.3	0.3	tr	tr	tr	nd	nd	nd	0.3	0.2	tr	tr	tr
11	910	906	2-Heptanol	tr	tr	nd	nd	nd	nd	tr	nd	nd	tr	nd	nd	nd	nd	tr	nd
12	990	989	6-Methyl-5-hepten-2-one	nd	nd	nd	0.6	0.5	tr	nd	nd	nd	nd	nd	0.7	0.4	tr	nd	nd
13	1004	997	6-Methyl-5-hepten-2-ol	nd	nd	nd	tr	0.8	nd	0.3	tr	nd	nd	nd	nd	1.1	nd	0.5	nd
14	1030	1031	Limonene	nd	nd	nd	nd	tr	tr	nd	nd	nd	nd	nd	nd	nd	nd	nd	nd
15	1050	1050	(*E*)-β-Ocimene	nd	nd	nd	0.5	nd	tr	nd	tr	nd	nd	nd	nd	nd	nd	nd	nd
16	1052	1050	2-Phenylethanal	nd	nd	nd	nd	0.3	nd	tr	nd	tr	tr	nd	tr	0.3	tr	tr	nd
17	1061	1052	Phenylmethanol	nd	nd	nd	nd	nd	nd	nd	nd	tr	0.4	nd	1.8	0.3	0.6	tr	0.9
18	1076	1076	1-Phenylethanone	0.3	nd	nd	nd	nd	nd	nd	nd	0.5	nd	nd	nd	nd	nd	nd	nd
19	1077	1075	*trans*-Linalool oxide (furanoid)	nd	0.5	nd	3.0	7.0	0.5	0.3	8.9	nd	2.2	nd	1.8	6.6	0.6	0.8	4.8
20	1083	1078	1-Phenylethanol	nd	nd	nd	0.2	nd	tr	nd	nd	0.8	2.1	nd	0.2	nd	tr	0.9	0.4
21	1093	1089	*cis*-Linalool oxide (furanoid)	nd	tr	nd	1.4	3.5	tr	tr	4.8	nd	1.0	nd	1.1	3.7	0.2	0.3	2.7
22	1100	1100	Undecane	0.2	0.6	nd	nd	1.2	nd	nd	0.6	nd	nd	nd	nd	nd	nd	nd	nd
23	1108	1104	Nonanal	0.3	0.3	0.2	6.5	3.7	3.4	0.4	2.1	0.4	0.9	nd	4.3	1.6	1.6	0.3	1.3
24	1111	1107	Linalool	0.2	3.0	0.6	2.6	6.7	9.1	1.9	8.9	1.0	7.9	tr	3.5	6.6	11.7	2.7	3.6
25	1114	1112	*cis*-Rose oxide	tr	nd	nd	nd	nd	nd	1.5	nd	nd	nd	nd	nd	nd	nd	1.4	nd
26	1129	1127	*trans*-Rose oxide	tr	nd	nd	nd	nd	nd	0.5	nd	nd	nd	nd	nd	nd	nd	0.3	nd
27	1134	1135	2-Phenylethanol	tr	nd	0.6	nd	nd	tr	nd	1.4	30.9	21.7	28.6	3.2	0.3	34.6	nd	64.4
28	1174	1170	1,4-Dimethoxybenzene	14.9	9.9	0.6	nd	0.1	2.9	nd	nd	8.6	17.0	0.3	tr	nd	1.3	nd	nd
29	1185	1176	1-Nonanol	nd	nd	nd	nd	nd	nd	nd	nd	nd	nd	nd	0.5	nd	nd	nd	nd
30	1187	1180	Myrtanal	0.3	tr	nd	0.4	nd	0.8	tr	tr	tr	tr	nd	nd	tr	1.0	tr	nd
31	1200	1200	Dodecane	0.3	0.3	nd	nd	tr	nd	nd	nd	nd	nd	nd	nd	0.2	nd	tr	nd
32	1206	1201	α-Terpineol	nd	0.5	nd	0.8	1.7	2.6	0.6	2.0	tr	1.6	nd	1.5	2.3	3.5	1.0	0.9
33	1225	1220	4,7-Dimethylbenzofuran	0.6	1.2	0.6	2.6	0.6	0.8	tr	0.9	0.2	0.7	nd	0.3	0.1	tr	nd	nd
34	1240	1232	Nerol	nd	0.5	nd	9.4	10.6	5.6	nd	2.0	nd	3.4	nd	11.6	14.9	9.9	nd	2.7
35	1242	1236	Citronellol	13.0	nd	6.1	nd	nd	nd	30.1	nd	30.7	nd	3.1	nd	nd	nd	44.8	nd
36	1254	1252	3-Phenylpropanol	nd	nd	nd	nd	nd	nd	nd	nd	nd	nd	nd	1.2	nd	nd	nd	nd
37	1267	1268	Geraniol	0.6	1.7	0.4	11.1	12.5	7.7	8.7	4.5	0.9	7.6	tr	15.4	19.8	12.5	15.1	4.4
38	1277	1271	Geranial	nd	nd	nd	0.5	tr	0.3	tr	nd	nd	tr	nd	0.6	0.3	0.4	tr	nd
39	1300	1300	Tridecane	0.9	0.8	nd	0.5	0.4	0.2	tr	tr	nd	nd	nd	nd	nd	nd	nd	nd
40	1329	1326	Methyl geranate	nd	nd	nd	0.9	tr	nd	0.3	nd	nd	nd	nd	2.2	0.3	nd	0.6	nd
41	1338	1331	Cinnamyl alcohol	nd	nd	nd	nd	nd	nd	nd	nd	nd	1.0	nd	11.0	nd	nd	nd	nd
42	1356	1356	Citronellyl acetate	tr	nd	tr	tr	nd	0.2	0.5	nd	nd	nd	nd	nd	nd	nd	0.4	nd
43	1367	1366	Neryl acetate	nd	nd	nd	tr	nd	nd	nd	nd	nd	nd	nd	tr	nd	nd	nd	nd
44	1375	1370	Eugenol	nd	nd	nd	tr	nd	nd	nd	nd	nd	nd	nd	0.5	nd	nd	nd	nd
45	1386	1384	Geranyl acetate	nd	nd	nd	0.7	tr	0.6	tr	0.5	nd	nd	nd	tr	tr	tr	tr	0.3
46	1392	1396	(*E*)-7-Tetradecene ^f^	nd	tr	nd	tr	nd	nd	nd	nd	nd	nd	nd	nd	nd	nd	nd	nd
47	1400	1400	Tetradecane	1.2	1.1	tr	0.6	0.9	1.2	nd	0.6	nd	nd	nd	nd	nd	nd	nd	nd
48	1403	1375	Geranic acid	nd	nd	nd	nd	nd	nd	nd	nd	0.7	nd	nd	3.6	4.3	2.8	7.6	0.9
49	1416	1410	Methyleugenol	nd	nd	nd	1.5	nd	nd	nd	nd	nd	nd	nd	1.3	tr	nd	nd	nd
50	1426	1418	1,3,5-Trimethoxybenzene	1.0	0.3	49.1	14.6	7.4	1.7	3.1	nd	1.2	0.6	64.7	18.0	21.3	6.3	6.6	tr
51	1457	1456	Geranyl acetone	nd	nd	nd	0.4	tr	nd	nd	nd	nd	nd	nd	0.4	tr	nd	nd	nd
52	1462	1462	2,6,10-Trimethyltridecane	tr	tr	nd	tr	0.4	0.6	0.2	1.0	nd	nd	nd	nd	nd	nd	nd	nd
53	1486	1484	Germacrene D	3.3	5.5	0.3	2.2	nd	0.9	tr	tr	nd	nd	nd	nd	nd	nd	nd	nd
54	1501	1500	Pentadecane	11.2	8.8	6.1	2.7	2.1	2.6	4.6	2.3	nd	nd	nd	nd	nd	nd	nd	nd
55	1512	1510	α-Farnesene	0.4	1.1	0.3	tr	tr	5.6	0.2	1.0	nd	nd	nd	nd	nd	nd	nd	nd
56	1528	1526	δ-Cadinene	tr	tr	nd	tr	nd	nd	nd	nd	nd	nd	nd	nd	nd	nd	nd	nd
57	1571	1570	3-Methylpentadecane	0.4	0.5	0.3	tr	nd	tr	nd	tr	nd	nd	nd	nd	nd	nd	nd	nd
58	1579	1578	(*Z*)-3-Hexadecene	0.2	0.6	tr	nd	nd	nd	nd	nd	nd	nd	nd	nd	nd	nd	nd	nd
59	1580	1580	(*Z*)-3-Hexenyl benzoate	nd	nd	nd	0.5	nd	tr	nd	tr	tr	nd	nd	tr	nd	nd	nd	nd
60	1600	1600	Hexadecane	0.4	1.2	0.3	0.3	0.7	1.2	0.3	1.1	nd	nd	nd	nd	nd	nd	nd	nd
61	1664	1666	2-Methylhexadecane	0.7	2.9	0.5	0.3	0.2	0.4	0.8	0.4	nd	nd	nd	nd	nd	nd	nd	nd
62	1671	1667	(*Z*)-9-Tetradecen-1-ol	8.4	8.5	9.2	1.6	0.3	0.8	1.5	1.5	nd	nd	nd	nd	nd	nd	nd	nd
63	1678	1677	(*E*)-8-Heptadecene	2.4	3.6	4.1	1.1	tr	tr	0.5	tr	nd	nd	nd	nd	nd	nd	nd	nd
64	1700	1700	Heptadecane	3.7	11.7	2.1	3.6	2.1	4.4	9.7	3.3	nd	0.2	nd	nd	tr	tr	tr	nd
65	1771	1771	3-Methylheptadecane	tr	0.9	nd	0.2	tr	0.3	0.2	0.3	nd	nd	nd	nd	nd	nd	nd	nd
66	1800	1800	Octadecane	0.2	0.6	0.4	0.2	0.3	0.8	0.6	0.5	nd	nd	nd	nd	nd	nd	nd	nd
67	1850	1849	Hexahydrofarnesyl acetone	nd	tr	nd	tr	tr	0.3	tr	0.7	nd	nd	nd	nd	nd	nd	nd	nd
68	1864	1864	2-Methyloctadecane	nd	0.2	nd	nd	nd	tr	0.2	nd	nd	nd	nd	nd	nd	nd	nd	nd
69	1874	1880	9-Nonadecene ^g^	0.4	0.6	0.4	1.9	0.1	0.4	0.5	tr	nd	nd	nd	nd	nd	nd	nd	nd
70	1900	1900	Nonadecane	4.0	9.7	2.1	4.6	4.8	8.4	13.0	7.3	nd	0.4	nd	tr	tr	tr	0.4	nd
71	1917	1909	2-Heptadecanol	nd	0.4	nd	nd	nd	nd	0.3	nd	nd	nd	nd	nd	nd	nd	nd	nd
72	1922	1919	Farnesyl acetone	nd	nd	nd	0.3	tr	nd	nd	nd	nd	nd	nd	nd	nd	nd	nd	nd
73	1972	1974	3-Methylnonadecane	0.3	0.4	tr	0.4	0.1	0.4	0.2	0.3	nd	nd	nd	nd	nd	nd	nd	nd
74	2000	2000	Eicosane	0.3	0.3	0.3	0.3	0.2	0.5	0.3	0.3	nd	0.5	nd	tr	nd	tr	nd	nd
75	2101	2100	Heneicosane	3.9	1.5	1.9	3.1	2.4	3.0	2.5	2.9	nd	nd	nd	nd	nd	nd	nd	nd
76	2125	2119	Phytol	0.1	tr	nd	0.3	nd	nd	nd	nd	nd	nd	nd	nd	nd	nd	nd	nd
77	2200	2200	Docosane	1.0	0.5	0.7	0.5	0.6	0.8	0.4	0.9	nd	nd	nd	nd	nd	nd	nd	nd
78	2230	2225	Eicosanal	nd	nd	nd	0.3	nd	tr	nd	nd	nd	nd	nd	nd	nd	nd	nd	nd
79	2273	2271	(*Z*)-9-Tricosene	0.2	nd	nd	0.3	nd	nd	nd	nd	nd	nd	nd	nd	nd	nd	nd	nd
80	2302	2300	Tricosane	9.3	6.0	5.9	3.6	7.5	10.9	4.2	14.2	nd	nd	nd	nd	nd	nd	nd	nd
81	2371	2371	3-Methyltricosane	1.6	0.7	0.8	0.5	0.9	1.6	0.3	1.3	nd	nd	nd	nd	nd	nd	nd	nd
82	2400	2400	Tetracosane	0.9	0.7	0.5	0.3	0.7	1.3	0.6	2.0	nd	nd	nd	nd	nd	nd	nd	nd
83	2501	2500	Pentacosane	3.8	2.2	2.2	2.0	3.5	6.6	2.6	9.1	nd	nd	nd	nd	nd	nd	nd	nd
84	2571	2567	3-Ethyltetracosane	1.1	0.6	0.5	0.3	0.6	1.4	0.5	1.5	nd	nd	nd	nd	nd	nd	nd	nd
85	2600	2600	Hexacosane	0.4	nd	tr	tr	tr	0.3	tr	0.3	nd	nd	nd	nd	nd	nd	nd	nd
			**Group composition (%)**																
			Hydrocarbons	52.9	63.7	29.8	32.2	31.8	55.4	43.0	52.4	nd	1.1	nd	0.1	0.4	0.1	0.5	nd
			Oxygenated compounds	45.5	34.8	69.6	65.5	67.8	42.9	56.2	46.7	99.4	98.6	99.8	98.3	99.2	99.6	99.3	99.7
			Non-terpene hydrocarbons	49.2	57.1	29.2	29.4	31.7	48.8	42.8	51.3	nd	1.1	nd	0.1	0.4	0.1	0.5	nd
			Terpene hydrocarbons	3.7	6.6	0.6	2.8	0.1	6.6	0.2	1.1	nd	nd	nd	nd	nd	nd	nd	nd
			Non-terpene oxygenated compounds	31.2	28.5	62.5	33.6	25.6	15.2	11.7	14.4	66.0	74.8	96.6	56.5	40.3	57.0	24.2	79.4
			Oxygenated terpenes	14.3	6.3	7.1	31.9	42.2	27.7	44.5	32.3	33.4	23.8	3.2	41.8	58.9	42.6	75.1	20.3
			Sum	98.4	98.5	99.4	97.7	99.6	98.3	99.2	99.1	99.4	99.7	99.8	98.4	99.6	99.7	99.8	99.7

^a^ Percentage compositions are mean value of triplicate extractions and duplicate injections (RSD < 5% for major components among three batches of extraction); ^b^ RI_exp_, experimental retention indices on HP-5 ms; ^c^ RI_lit_, retention indices from literature; ^d^ nd, not detected under analytical conditions used; ^e^ tr, trace (<0.05%); ^f^ (*E*)-7-Tetradecene, exact isomer not specified: (*E*)-7-tetradecene, (*E*)-6-tetradecene, (*E*)-3-tetradecene or (*E*)-4-tetradecene; ^g^ 9-Nonadecene, (*E*/*Z*) isomer not specified.

**Table 3:** Results of quantitation towards major oxygenated components in hydrolate extracts of *Paeonia suffruticosa* Andr. cultivars.

Components	Contents in Hydrolate Extracts (mg/g) ^a^							
	JYH	JXQ	SHT	FDB	HH	YH	BXT	WLPS
2-Phenylethanol	286.3	198.4	259.7	28.6	3.4	319.5	nd ^b^	590.1
1,3,5-Trimethoxybenzene	9.6	5.2	596.4	165.1	194.5	59.0	58.7	1.5
Geraniol	7.8	68.3	1.2	139.7	180.6	113.7	141.2	39.8
Citronellol	280.1	nd	27.5	nd	nd	nd	410.6	nd

^a^ Contents are mean value of triplicate extractions and duplicate injections (RSD < 5% among three batches of extraction for components the contents of which were higher than 20 mg/g); ^b^ nd, not detected under analytical conditions used.

## Supplementary Materials

The following are available online, Figure S1: Mass spectra (GC-MS, 70 eV) of representative oxygenated compounds from hydrolate extracts of eight *Paeonia suffruticosa* Andr. cultivars: (**a**) 2-phenylethanol; (**b**) geraniol; (**c**) citronellol; (**d**) 1,3,5-trimethoxybenzene; (**e**) (*Z*)-3-hexen-1-ol.
