# Supplementary material for: Comparative Chemical Profiles of Essential Oils and Hydrolate Extracts from Fresh Flowers of Eight Paeonia suffruticosa Andr. Cultivars from Central China
Source: Molecules. 2018 Dec 10;23(12):3268. doi: 10.3390/molecules23123268 (PMC6321490; doi:10.3390/molecules23123268)
Supplement: Supplementary file 1 [file molecules-23-03268-s001.pdf]

Supplementary Materials

Comparative Chemical Profiles of Essential Oils and Hydrolate Extracts from Fresh Flowers of Eight *Paeonia suffruticosa* Andr. Cultivars from Central China

Gaoming Lei [1,*], Jie Li [1], Tao Zheng [2], Junqiao Yao [3], Jingjing Chen [1] and Lengxin Duan [1]

[1] Department of Pharmaceutical Sciences, Medical College, Henan University of Science and Technology, Luoyang 471023, China; lijie@haust.edu.cn (J.L.); 170317200708@stu.haust.edu.cn (J.C.); lengxinduan@haust.edu.cn (L.D.)

[2] National Flower Garden Administration of Luoyang, Luoyang 471022, China; tony7811@163.com

[3] Department of Scientific Research, Peony Institute of Luoyang, Luoyang 471022, China; mdsyjq@163.com

* Correspondence: leigm@haust.edu.cn; Tel.: +86-187-3630-3548

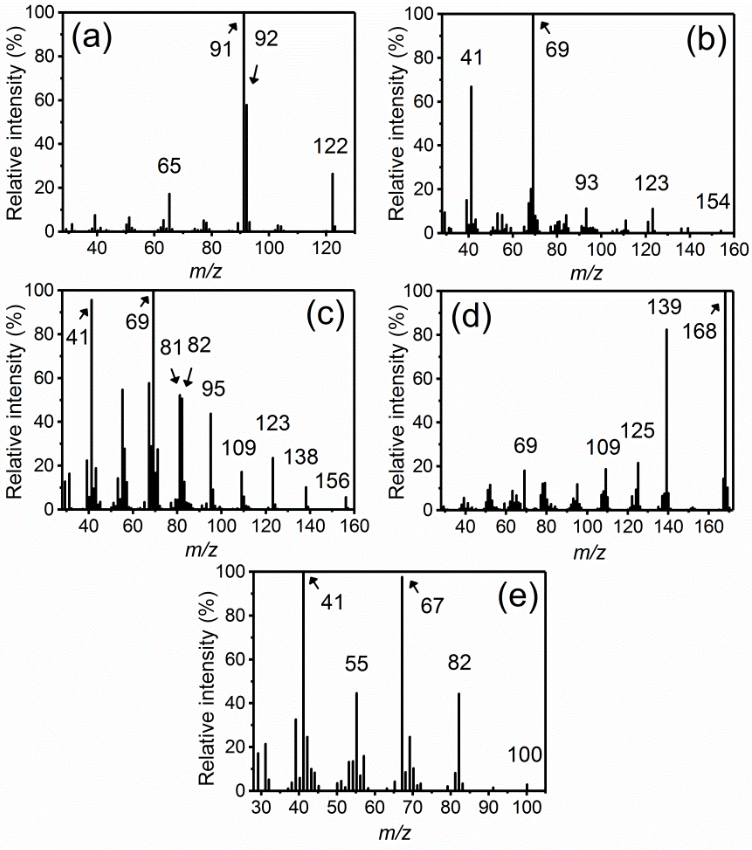

Figure S1. Mass spectra (GC-MS, 70 eV) of representative oxygenated compounds from hydrolate extracts of eight *Paeonia suffruticosa* Andr. cultivars: (**a**) 2-phenylethanol; (**b**) geraniol; (**c**) citronellol; (**d**) 1,3,5-trimethoxybenzene; (**e**) (*Z*)-3-hexen-1-ol.
